# Experimental Observations on the Structural Transitions and Metallization in the Two-Dimensional Layered Compound of Cadmium Phosphide Sulfide Under Extreme Conditions

**DOI:** 10.3390/molecules31162760

**Published:** 2026-08-08

**Authors:** Xinyu Zhang, Lidong Dai, Haiying Hu, Ziqiang Xu, Juxiang Shao, Hongchun Luo, Jiajun Zhu, Zhongying Mi, Tao Wang, Miao Ren, Yu Gao, Meiling Hong

**Affiliations:** 1 Key Laboratory of Computational Physics of Sichuan Province, College of Mathematics and Physics, Yibin University, Yibin 644007, China; zhangxinyu181@mails.ucas.ac.cn (X.Z.);; 2 School of Physics and Electronic Science, Guizhou Normal University, Guiyang 550025, China; 3 Key Laboratory of High-Temperature and High-Pressure Study of the Earth’s Interior, Institute of Geochemistry, Chinese Academy of Sciences, Guiyang 550081, China

**Keywords:** CdPS_3_, phase transitions, metallization, high pressure, hydrostaticity

## Abstract

Cadmium phosphorous trisulfide (CdPS_3_), a prototypical member of metal thio- and selenophosphates (MTPs), has garnered tremendous research interest due to its fundamental properties and potential for novel applications. In this paper, we conducted a comprehensive investigation on the structural evolution and electrical transport behaviors of CdPS_3_ up to 59.1 GPa under different hydrostatic environments by means of Raman spectroscopy and electrical conductivity measurements. Under non-hydrostatic compression, CdPS_3_ experienced a succession of structural modifications from C2/m to *R*$\bar{3}$ phases at 1.4(5) GPa, then to the isostructural *R*$\bar{3}$ phase at 7.8(5) GPa and sequentially to the *P*$\bar{3}1$*m* phase at 29.8(11) GPa, followed by a semiconductor-to-metal transition at 51.3(8) GPa. Under hydrostatic pressurization, ~2.0 GPa pressure hysteresis for the *R*$\bar{3}$-to-*P*$\bar{3}1$*m* structural modification and metallization was identified, which can be reasonably interpreted by the impact of deviatoric stress. Upon decompression, the structural transitions of CdPS_3_ were demonstrated to be reversible with the existence of considerable pressure hysteresis under different hydrostatic environments. Our findings on CdPS_3_ not only lay a solid foundation for exploring the physicochemical properties of other MTPs under extreme conditions but also push forward its applications in high-performance multifunctional devices.

## 1. Introduction

In recent decades, a large family of MTPs with the general chemical formula of MPX_3_ (M = V, Mn, Fe, Co, Ni, Zn, Cd, Sn, Pb; X = S, Se) have stimulated prominent interest from the scientific community as multifunctional van der Waals (vdW) compounds, searching for the development of potential applications in sensors, spintronics, optoelectronics and energy-storage devices [1,2,3]. Unlike the antiferromagnetic Mott insulators (e.g., VPS_3_, MnPS_3_, FePS_3_, CoPS_3_, etc.) possessing partially filled *d* orbitals, CdPS_3_ is a diamagnet with fully filled *d* orbitals (4*d*^10^ configuration) [4,5]. At atmospheric pressure, CdPS_3_ crystallizes into the monoclinic *C*2/*m* symmetry and belongs to a wide-bandgap semiconductor (*E*_g_ = ~3.4 eV). In the layered structure of CdPS_3_, Cd^2+^ cations are coordinated to [P_2_S_6_]^4−^ polyanions, forming the distorted hexagonal network of edge-sharing octahedra, and the layers are held together by vdW interactions along the *c* axis [4,6,7,8].

The interatomic distance, crystalline structure, and magnetic and electronic configurations of MPX_3_ can be effectively manipulated via the application of external pressure, which belongs to a clean and controllable physical method without composition alteration [4,6,9,10,11,12,13,14,15,16,17,18,19,20,21]. Encouragingly, a wealth of exotic phenomena such as piezochromism, interlayer sliding, volume collapse, structural transition, spin crossover, metallization and superconductivity emerged in pressurized MPX_3_ [4,6,9,10,11,12,13,14,15,16,17,18,19,20,21]. Despite the extensive high-pressure studies on CdPS_3_, several critical issues remain unresolved, including the controversies over the structural transition pressures and sequences along with the lack of direct experimental evidence for metallization [6,19,20]. Preliminary theoretical calculations on CdPS_3_ predicted a sequence of structural transformations from *C*2/*m* to *R*$\bar{3}$ phases (1.0 GPa), then to an isostructural phase transition (IPT) (6.0 GPa), and ultimately to a *P*$\bar{3}1$*m* phase (25.0 GPa), which was corroborated by high-pressure Raman scattering results [6]. Subsequent theoretical calculations reproduced the *C*2/*m*-to-*R*$\bar{3}$ structural transition at 1.5 GPa and predicted the metallicity of the *P*$\bar{3}1$*m* phase at 25.0 GPa [20]. Although the pressure-induced bandgap closure has been predicted in CdPS_3_, it has not been supported by experimental evidence. Recent synchrotron infrared absorbance, Raman spectroscopy and lattice dynamics calculations reported that CdPS_3_ endured the *C*2/*m*-to-*R*$\bar{3}$ structural transition at 10.0 GPa and sequentially transformed into the *P*3 phase at 18.0 GPa, followed by two structural transitions at the respective pressures of 29.0 and 33.0 GPa with the preservation of semiconducting characteristics up to 35.0 GPa [19]. Therefore, the *C*2/*m*-to-*R*$\bar{3}$ transition pressure (ranging from 1.0 to 10.0 GPa) and the subsequent transition of *R*$\bar{3}$ to *P*$\bar{3}1$*m* or *P*3 phases at higher pressure remain debated.

On the other hand, Cui et al. reported that the metallization of NiPS_3_ was highly sensitive to the strain and hydrostatic environment with the significant pressure hysteresis of ~17.0 GPa under the quasi-hydrostatic condition compared to that under the non-hydrostatic conditions, similarly to our previous studies in other MTPs (e.g., ZnPS_3_, CuCrP_2_S_6_, CuInP_2_S_6_, CuInP_2_Se_6_, etc.) [22,23,24,25,26]. As for CdPS_3_, the influence of hydrostaticity on its high-pressure behaviors is yet to be clarified. In previous high-pressure experiments of CdPS_3_, silicon oil, KBr and petroleum jelly were used as the pressure-transmitting mediums (PTMs), which lose hydrostaticity at lower pressure. In comparison with the solid and liquid PTMs, the noble gases (e.g., helium, neon, argon, etc.) could provide a more homogenous pressure environment and thus are considered to be better PTMs [27,28]. Therefore, it is imperative to perform the high-pressure experiments of CdPS_3_ under different hydrostatic environments so as to illustrate the possible influence of hydrostaticity degree.

In this article, we filled in these research blanks and explored the high-pressure structural, vibrational and electrical transport properties of CdPS_3_ under different hydrostatic environments by virtue of a diamond anvil cell (DAC) in conjunction with Raman scattering and electrical conductivity measurements. Based on our and previous results, a comprehensive phase diagram of the MPS_3_ family (M = Mn, Fe, Co, Ni, Zn and Cd) was mapped out under extreme conditions.

## 2. Results and Discussion

### 2.1. High-Pressure Raman Spectroscopy Results

**Figure 1:**
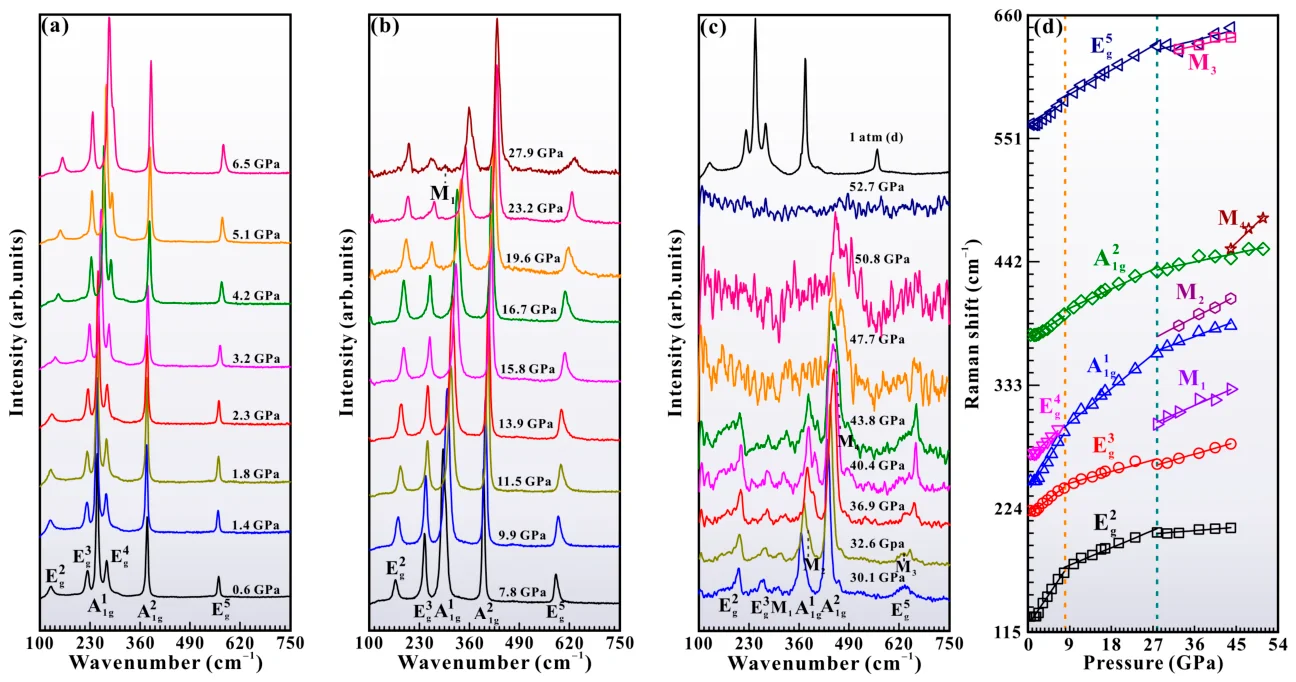
High-pressure Raman spectroscopic results of CdPS_3_ under the non-hydrostatic condition. (**a**–**c**) The results upon applying pressure from 0.6 to 52.7 GPa and the recovered sample. Herein, the decompression process is marked by the symbol d. (**d**) Pressure dependence of Raman shift relations. The errors in Raman shifts are smaller than the size of symbols.

Figure 1a–c show the representative Raman spectroscopic results of CdPS_3_ when applying pressure from 0.6 to 52.7 GPa and the recovered sample under the non-hydrostatic condition. At 0.6 GPa, six well-defined Raman peaks located at 127.9, 222.0, 248.2, 272.7, 377.6 and 563.5 cm^−1^ are discernible within the wavenumber range of 100–750 cm^−1^, which are assigned to the $E_{g}^{2}$, $E_{g}^{3}$, $A_{1g}^{1}$, $E_{g}^{4}$, $A_{1g}^{2}$ and $E_{g}^{5}$ modes of CdPS_3_. The $E_{g}^{2}$, $E_{g}^{3}$, $E_{g}^{4}$ and $E_{g}^{5}$ modes arise from the in-plane vibrations of [P_2_S_6_]^4−^ units, whilst the $A_{1g}^{1}$ and $A_{1g}^{2}$ modes correspond to their out-of-plane vibrations [6,8,19].

**Figure 2:**
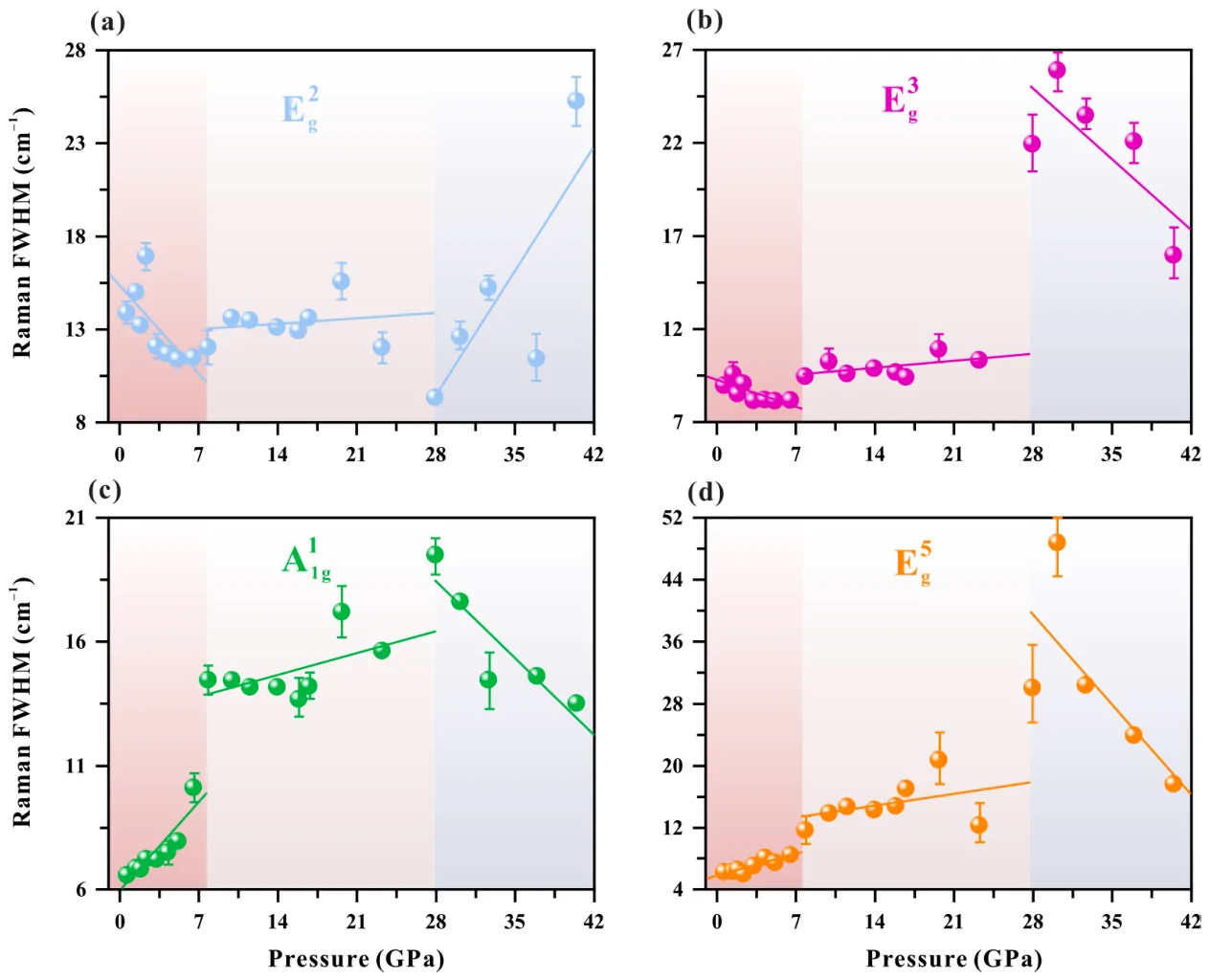
Pressure-dependent Raman FWHM relations for (**a**) $E_{g}^{2}$, (**b**) $E_{g}^{3}$, (**c**) $A_{g}^{1}$ and (**d**) $E_{g}^{5}$ modes under the non-hydrostatic condition. The errors in Raman FWHMs are within the size of the symbols.

From Figure 1a–c, all Raman modes of the specimen showed a universal trend with considerable blueshift under compression, which originated from the contracted bond length and the strengthened bond strength. Below 6.5 GPa, the $E_{g}^{2}$ peak intensity substantially attenuated as the pressure rose. Notably, the $A_{1g}^{1}$ and $E_{g}^{4}$ modes gradually approached each other with increasing pressure and completely overlapped at 7.8 GPa. When the pressure exceeded 7.8 GPa, the $E_{g}^{2}$ and $A_{1g}^{2}$ modes progressively intensified, concomitantly with the appreciable suppression and broadening of $A_{1g}^{1}$ and $E_{g}^{5}$ modes. Beyond 11.5 GPa, the $A_{1g}^{2}\;$ mode gradually evolved into the dominant peak of the sample, whilst the $E_{g}^{3}$ mode asymmetrically broadened after 19.6 GPa. As the pressure reached 27.9 GPa, a faint Raman peak at 298.4 cm^−1^ (labeled as the M_1_ mode) emerged on the right side of the $E_{g}^{3}$ mode, followed by the asymmetry of the $A_{1g}^{1}$ mode and the appearance of a tiny shoulder located at 385.5 cm^−1^ (designated as the M_2_ mode) at 32.6 GPa. Simultaneously, a weak Raman peak at 630.2 cm^−1^ (labeled as the M_3_ mode) appeared on the left side of the $E_{g}^{5}$ mode. With further pressurization to 43.8 GPa, the $A_{1g}^{2}$ mode split and a new Raman peak at the position of 453.4 cm^−1^ (marked as the M_4_ mode) emerged. Beyond 43.8 GPa, all Raman peaks of CdPS_3_ weakened and ultimately became featureless at 52.7 GPa, signaling the gradual reduction in bandgap and eventual metallization, as reported in other MTPs (e.g., ZnPS_3_, CuCrP_2_S_6_, CuInP_2_S_6_, CuInP_2_Se_6_, etc.) [23,24,25,26]. In addition, some high-pressure Raman spectra of CdPS_3_ have a higher signal-to-noise ratio (SNR) than others, which is probably related to the following causes: (i) The high-pressure Raman spectra of CdPS_3_ are not collected from the same spot of the sample chamber due to the inevitable sample movement under compression, which may lead to the discrepancy in SNR [29]. (ii) Generally speaking, the sample became thinner with increasing pressure, which may also result in the different SNRs for the collected high-pressure Raman spectra of CdPS_3_. To further illuminate the high-pressure phase stability of CdPS_3_, the evolution of Raman shifts and full width at half-maximums (FWHMs) against pressure under the non-hydrostatic condition are visualized in Figure 1d and Figure 2, and further, their corresponding linear fitting results (d*ω*/d*P* and d*F*/d*P*, unit: cm^−1^ GPa^−1^; the symbols of *ω*, *F* and *P* stand for Raman shift, FWHM and pressure, respectively) are tabulated in Tables S1 and S2. As can be seen from Table S1, the $A_{1g}^{1}$ mode stemming from the out-of-plane bending of [P_2_S_6_]^4−^ units exhibits a larger pressure coefficient than those of $E_{g}^{3}$, $E_{g}^{4}$ and $E_{g}^{5}$ modes, suggesting that the out-of-plane vibrations are more sensitive to pressure than the in-plane vibrations. Similar situations of anisotropic compressibility have been extensively reported in other MTPs (e.g., CoPS_3_, ZnPS_3_, SnPSe_3_, etc.) [9,12,21,26]. Furthermore, the Raman shifts of $E_{g}^{2}$, $E_{g}^{3}$, $A_{1g}^{1}$, $A_{1g}^{2}$ and $E_{g}^{5}$ modes along with the FWHMs for $E_{g}^{2}$, $E_{g}^{3}$, $A_{1g}^{1}$ and $E_{g}^{5}$ modes presented two remarkable discontinuities at 7.8(3) and 27.9(6) GPa under the non-hydrostatic condition, which was presumably linked to the occurrence of structural modifications in CdPS_3_.

Figures S1 and S2 show the high-pressure Raman spectral evolution of CdPS_3_ under the hydrostatic condition, together with the pressure dependence of Raman shifts and FWHMs. On the whole, the high-pressure Raman spectra of CdPS_3_ and the resultant pressure-dependent Raman shifts and FWHMs under the hydrostatic condition bore resemblance to those under the non-hydrostatic condition. Specifically, CdPS_3_ possibly underwent two successive phase transitions at 7.3(4) and 29.3(8) GPa, followed by a metallization at 53.9(8) GPa under the hydrostatic condition. By comparison, we found that the first phase transition of CdPS_3_ occurred at analogous pressures (i.e., 7.8(3) and 7.3(4) GPa under non-hydrostatic and hydrostatic conditions), which was likely attributed to the negligible deviatoric stress in the sample chamber of the DAC below ~10.0 GPa. Nevertheless, the second phase transition and metallization of CdPS_3_ were delayed by ~2.0 GPa under the hydrostatic condition compared to under the non-hydrostatic condition. Analogous observations were reported in previous studies on other MTPs (e.g., ZnPS_3_, CuCrP_2_S_6_, CuInP_2_S_6_, CuInP_2_Se_6_, etc.) and commonly attributed to the impact of deviatoric stress [23,24,25,26]. Beyond ~10.0 GPa, the deviatoric stress in the non-hydrostatic condition enhances more quickly than that in the hydrostatic condition, promoting the appearance of a second phase transition and metallicity in CdPS_3_. As presented in Figure 1c and Figure S1c, after complete unloading, the Raman spectra reverted back to their initial profiles and positions with some weak and broad Raman peaks, demonstrating that the structural transitions of CdPS_3_ were reversible with the existence of residual strain in the decompressed specimens under different hydrostatic environments. In short, our high-pressure Raman scattering results revealed that CdPS_3_ presumably underwent two phase transitions at 7.8(3) and 27.9(6) GPa and then a metallization at 52.7(8) GPa under the non-hydrostatic condition. Furthermore, the second phase transition and metallization were postponed by ~2.0 GPa under the hydrostatic condition owing to the impact of deviatoric stress.

In fact, Niu et al. and Shah et al. reported the high-pressure phase stability of CdPS_3_ single crystals at pressures up to 15.5 and 35.0 GPa using Raman spectroscopy [6,19]. Overall, our high-pressure Raman spectra of CdPS_3_ were akin to previously reported results. Specifically, the merging of $A_{1g}^{1}$ and $E_{g}^{4}$ Raman peaks was commonly observed in both our and previous results at 7.8–10.2 GPa, which was probably associated with the occurrence of IPT. Previous studies reported the asymmetry of the $A_{g}^{3}$ Raman peak at 18.4 GPa and the emergence of a new Raman peak at 29.4 GPa, which was detected at similar pressures of 19.6 and 27.9 GPa in our work. Previous theoretical calculations predicted the appearance of the *P*$\bar{3}1$*m* phase in CdPS_3_ at ~25.0 GPa [6,20]. This value is close to our experimentally determined transition pressure of 27.9(6) GPa, which was deduced from the emergence of the M_1_ mode and the prominent discontinuities in Raman shifts and FWHMs of $E_{g}^{2}$, $E_{g}^{3}$, $A_{1g}^{1}$, $A_{1g}^{2}$ and $E_{g}^{5}$ modes. Shah et al. reported the splitting of the $A_{g}^{7}$ Raman peak into two separate components at 32.8 GPa and we observed the same feature at a comparable pressure of 32.6 GPa [19]. Despite the similarity in Raman spectral changes of CdPS_3_ under compression, the inconsistent transformation pressures were acquired between our work and previous studies [19]. Specifically, our Raman scattering results revealed two structural transitions at 7.8(3) and 27.9(6) GPa under the non-hydrostatic condition. By contrast, Shah et al. reported the phase transitions at ~18.0 and ~33.0 GPa, which likely correspond to the initiation of phase transition and metallization rather than the completion of these phase changes [19].

Niu et al. claimed the *C*2/*m*-to-*R*$\bar{3}$ phase transition took place at ~0.1 GPa, as evidenced by the appearance of a new Raman peak at ~30 cm^−1^ and the disappearance of a Raman peak at ~80 cm^−1^ [6]. However, Shah et al. detected the Raman peaks at ~30 and ~80 cm^−1^ under ambient conditions and proposed that the *C*2/*m* and *R*$\bar{3}$ phases are structurally similar [19]. To resolve the above-mentioned contradiction between Niu et al. and Shah et al., we measured the high-pressure Raman spectra of CdPS_3_ under the non-hydrostatic condition using a 785.0 nm excitation source, as presented in Figure S3. Only a sharp Raman peak at 80.9 cm^−1^ was observed under ambient conditions, which arises from the dimerization of cadmium atoms and is assigned to the $E_{g}^{1}$ mode of CdPS_3_ [6,8,19]. Notably, a faint Raman peak located at 45.9 cm^−1^ (denoted as the $B_{g}^{1}$ mode) became visible at 0.9 GPa, which presumably originates from the rigid layer compressional mode [30]. At 0.9–7.2 GPa, the $B_{g}^{1}$ mode progressively intensified with applied pressure, accompanied by the substantial decline in the $E_{g}^{1}$ mode. Above 7.8 GPa, the $E_{g}^{1}$ mode gradually strengthened with increasing pressure till the maximum value of 24.8 GPa. In addition, the $B_{g}^{1}$ mode presented a redshift with a slope of –0.33 cm^−1^ GPa^−1^ at 0.9–7.2 GPa and thereafter moved towards higher frequencies at a slope of 0.60 cm^−1^ GPa^−1^ beyond 7.8 GPa. Consequently, the appearance of the $B_{g}^{1}$ mode and its redshift-to-blueshift inversion at 0.9(2) GPa offered definitive evidence for the appearance of the *C*2/*m*-to-*R*$\bar{3}$ phase transition in CdPS_3_. Nevertheless, the Raman peak at ~30 cm^−1^, deriving from the buckling of the cadmium honeycomb lattice, was not observed in our work, which may be attributed to the discrepancy in experimental samples [19,30]. Our starting materials were high-quality CdPS_3_ powders with the grain size of ~10 µm (see the inset of Figure 7b), which was synthesized by heating the stoichiometric proportions of cadmium, phosphorus and sulfur powders in a quartz glass ampule. However, centimeter-sized CdPS_3_ single crystals (with the chemical formula of Cd_1.07_PS_2.96_) prepared by the chemical vapor transport (CVT) method were used in prior investigations [6,19].

### 2.2. High-Pressure Electrical Conductivity Results

**Figure 3:**
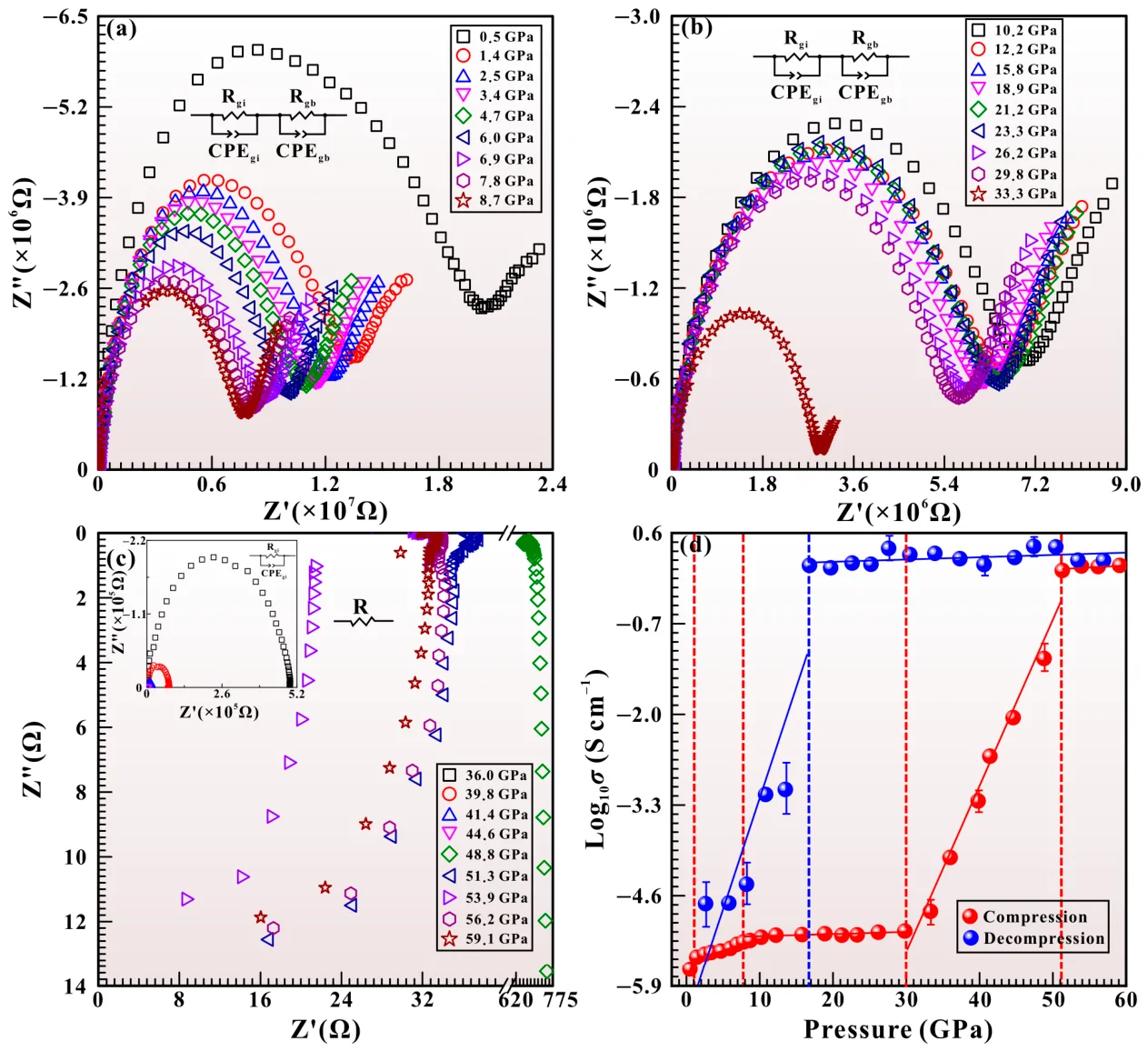
High-pressure Nyquist diagrams of impedance spectra on CdPS_3_. (**a**–**c**) The results under the conditions of 0.5–59.1 GPa and room temperature. (**d**) The variation in logarithmic electrical conductivity under high pressure in the processes of compression and decompression. The errors in electrical conductivities are within the size of the symbols.

Figure 3a–c illustrate the Cole–Cole diagrams of CdPS_3_ within the pressure range of 0.5–59.1 GPa and at room temperature. Regular semicircular arcs at high frequency plus inclined lines at low frequency are seen in the impedance spectra at 0.5–33.3 GPa, corresponding to the grain interior and grain boundary responses of CdPS_3_. As the pressure surpassed 36.0 GPa, the oblique straight lines disappeared and only semicircular arcs were visible throughout the entire frequency regime, indicating that the grain interior contribution dominated the electrical transport process. With further compression to 48.8 GPa, a tilted curve perpendicular to the real axis was recognized in the fourth quadrant. By fitting the gathered impedance spectra using suitable equivalent electric circuits with the help of ZView 3.5 software, the electrical resistances (*R*) of CdPS_3_ at high pressures were accurately determined. The impedance spectra in the pressure range of 0.5–33.3 GPa could be well fitted by R_gi_–CPE_gi_ and R_gb_–CPE_gb_ circuits in series, where R_gi_ and CPE_gi_ are the resistor and constant-phase element of the grain interior, and R_gb_ and CPE_gb_ represent the resistor and constant-phase element of the grain boundary, respectively. A single R_gi_–CPE_gi_ circuit was applied to simulate the semicircular arcs between 36.0 and 44.6 GPa. When the pressure exceeded 48.8 GPa, an individual resistor (R) was employed to fit the oblique lines. After that, the electrical conductivity (*σ*) of CdPS_3_ was calculated using the following formula:

*σ* = *L*/*SR* (1)

wherein *R* denotes resistance, and *S* and *L* represent the cross-sectional area of electrodes (cm^2^) and the distance between electrodes (cm), respectively. Figure 3d shows the logarithmic electrical conductivity of CdPS_3_ as a function of pressure in the processes of compression and decompression. A steep augmentation of electrical conductivity was identified as the pressure was raised from 0.5 to 1.4 GPa. This phenomenon may originate from (i) the *C*2/*m*-to-*R*$\bar{3}$ structural transition [6,20] or (ii) the compaction of pores and grain boundaries due to the powder nature of the experimental sample. When the applied pressure was enhanced from 1.4 to 7.8 GPa, the electrical conductivity exhibited a gentle upturn with a rate of 0.033 S cm^−1^ GPa^−1^, followed by the almost saturated conductivity with a tiny slope of 0.0057 S cm^−1^ GPa^−1^ ranging from 7.8 to 29.8 GPa. As the pressure was elevated from 29.8 to 51.3 GPa, the electrical conductivity of the sample was exceptionally sensitive to pressure with a larger slope of 0.24 S cm^−1^ GPa^−1^. Remarkably, the high electrical conductivity value of ~0.13 S cm^−1^ beyond 51.3 GPa and the flat pressure-dependent conductivity relation with a faint slope of 0.0076 S cm^−1^ GPa^−1^ are the representative manifestations of metallicity in pressurized CdPS_3_. As a matter of fact, the pressure-driven metallization has been widely reported in other MTPs (e.g., ZnPS_3_, FePS_3_, NiPS_3_, MnPS_3_, Sn_2_P_2_S_6_, Sn_2_P_2_Se_6_, Pb_2_P_2_S_6_, etc.) [10,11,13,14,15,16,17,18,26]. Upon depressurization, the electrical conductivity of the specimen remained nearly invariant until 16.8 GPa. Upon further release of pressure below 16.8 GPa, the electrical conductivity showed a prominent decline by more than four orders of magnitude at a large slope of 0.32 S cm^−1^ GPa^−1^. Additionally, we found that the electrical conductivity of the decompressed sample was approximately one order of magnitude higher than that of the starting sample, which was possibly associated with the reduction in grain size caused by the compression–decompression cycle [31]. Despite some discrepancies in the magnitudes of electrical conductivity between the pristine and depressurized samples, the metallization of CdPS_3_ was found to be reversible with a pronounced pressure hysteresis of ~38.0 GPa upon pressurization and depressurization.

**Figure 4:**
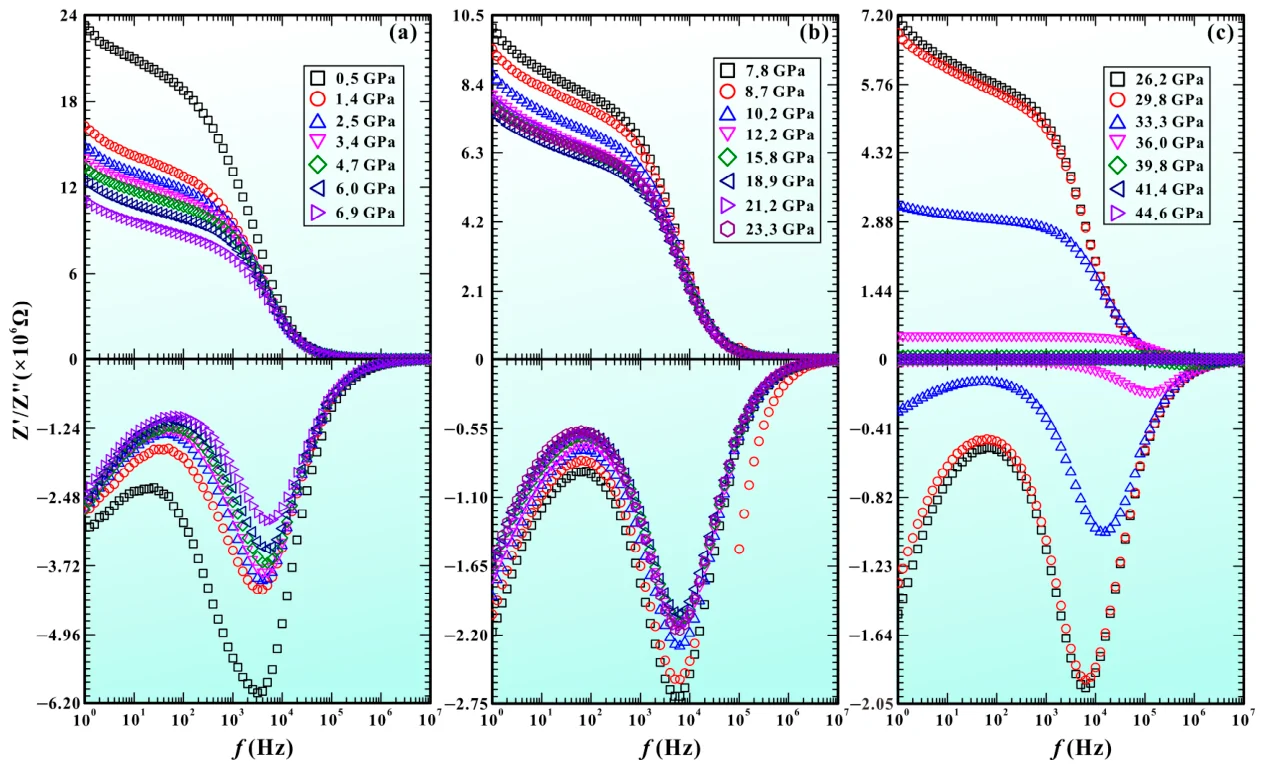
Bode plots of impedance spectra on CdPS_3_ under high pressure. (**a**–**c**) Frequency dependence of real and imaginary parts of impedance at the respective pressure ranges of 0.5–6.9 GPa, 7.8–22.3 GPa and 26.2–44.6 GPa. Herein, the y axis of Z′/Z″ denotes the ratio of real and imaginary parts of impedance.

Figure 4 depicts the high-pressure Bode plots of CdPS_3_, illustrating the evolution of the real (Z′) and imaginary (Z″) components of impedance with respect to frequency at diverse pressures. The Z′ dramatically diminished as the frequency was increased from 10^0^ to 10^5^ Hz and then stabilized at a constant frequency beyond 10^5^ Hz, elucidating the augmentation of electrical conductivity caused by the decline in the energy barrier and the release of charge carriers. By contrast, with ascending frequency, the Z″ showed a smooth rise and sequential declined to a minimum value termed as the characteristic relaxation frequency (*f*_min_), followed by a rapid boost. The height of the relaxation peak dropped and the *f*_min_ moved to higher frequencies under compression, which can be interpreted by the short polarization process. The corresponding variation in relaxation frequency against pressure was displayed in Figure S4. With the application of pressure, a steep rise in relaxation frequency with a rate of 0.11 Hz GPa^−1^ was attained below 1.4 GPa, followed by a mild increment at a slope of 0.016 Hz GPa^−1^ between 1.4 and 7.8 GPa. Subsequently, in the pressure range of 7.8–29.8 GPa, the relaxation frequency of the specimen leveled off at a minor slope of 0.0023 Hz GPa^−1^. When the pressure exceeded 29.8 GPa, a sudden upturn in relaxation frequency with a large rate of 0.19 Hz GPa^−1^ was discernible.

The pressure dependence of activation energy (d*H*/d*P*, unit: eV GPa^−1^) for CdPS_3_ was deduced from the following equation, and the corresponding activation energies at different pressure regimes are given in Table S3.

d(ln *f*)/d*P* = −(1/*k*_B_
*T*) (d*H*/d*P*) (2)

Herein, the symbols of *f*, *P*, *k*_B_, *T* and *H* denote the relaxation frequency (Hz), pressure (GPa), Boltzmann constant, temperature (K) and activation energy (eV). As illustrated in Table S3, the activation energies of CdPS_3_ were determined to be −6.48, −0.94, −0.10 and −12.83 meV GPa^−1^ at the respective pressure ranges of 0.5–1.4 GPa, 1.4–7.8 GPa, 7.8–29.8 GPa and 29.8–44.6 GPa. As the pressure was loaded from 1.4 GPa through 7.8 GPa to 29.8 GPa, the activation energies of the specimen increased significantly, implying that the charge carrier transfer became more difficult. Nevertheless, the dramatic reduction in activation energies beyond 29.8 GPa demonstrated that the carrier transport became easier. In summary, the conspicuous discontinuities in electrical conductivity and relaxation frequency at 1.4(5), 7.8(5) and 29.8(11) GPa may be attributed to the structural transformations of CdPS_3_, followed by a metallization at 51.3(8) GPa.

### 2.3. Variable-Temperature Electrical Conductivity Results

**Figure 5:**
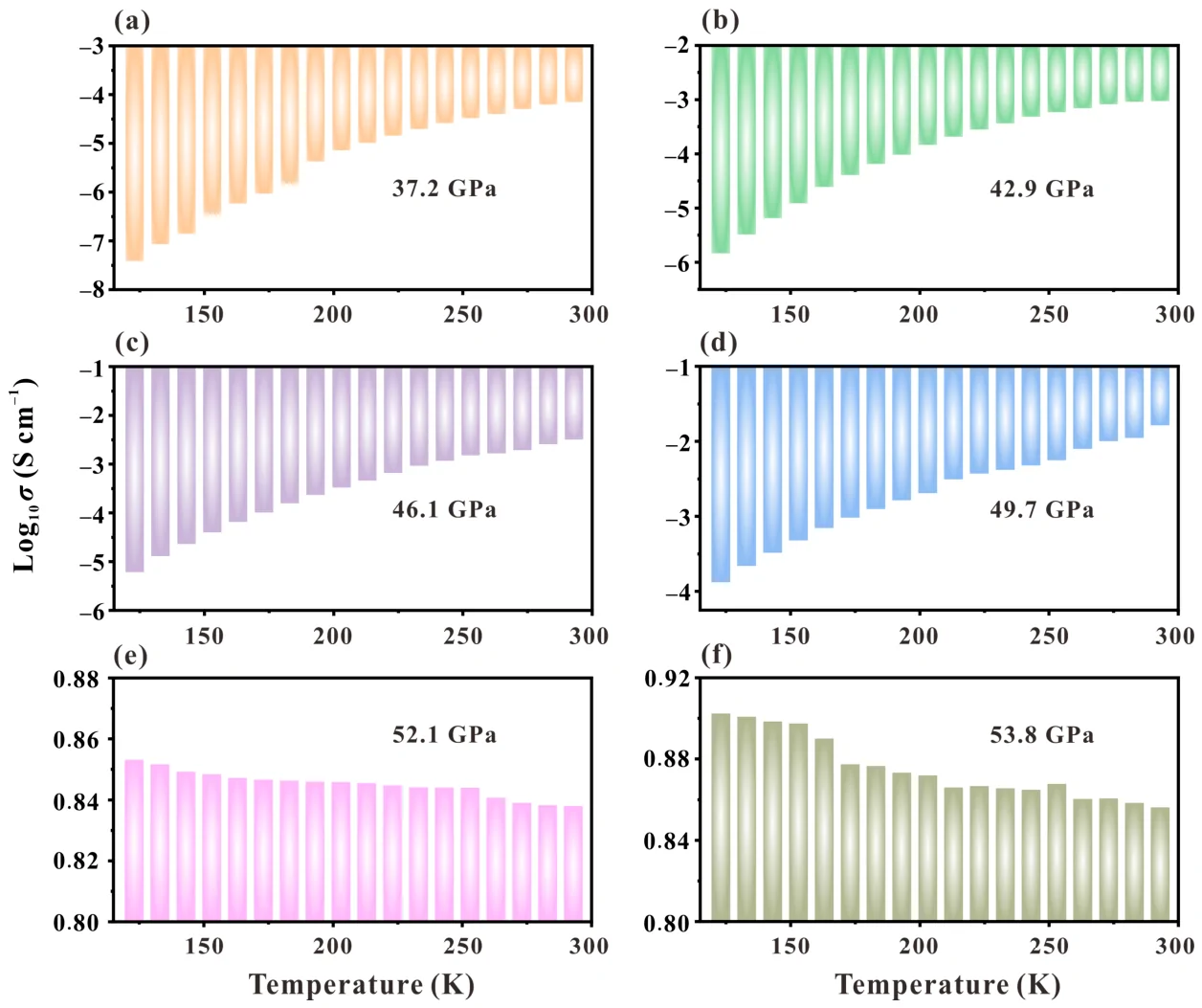
Variable-temperature electrical conductivity results on CdPS_3_ upon pressurization. (**a**–**d**) The semiconducting state of the specimen at 37.2, 42.9, 46.1 and 49.7 GPa, respectively. (**e**,**f**) The metallic character of CdPS_3_ at the respective pressures of 52.1 and 53.8 GPa.

To validate the metallicity in compressed CdPS_3_, a series of low-temperature electrical conductivity measurements were performed at 37.2, 42.9, 46.1, 49.7, 52.1 and 53.8 GPa. Based on the classical solid-state physics theory, a semiconductor features a positive temperature-dependent electrical conductivity relation driven by the thermal activation of charge carriers, whereas metal is characterized by a negative temperature-dependent electrical conductivity relation as a consequence of the strengthened electron–phonon scattering [23,24,25,26,32,33,34,35,36]. As evident from Figure 5, the upturn in conductivity with rising temperature below 49.7 GPa corroborated the semiconducting behavior of the specimen. At 52.1 GPa, the electrical conductivity presented a declining tendency at elevated temperature, indicative of the metallic character in CdPS_3_. In addition, the activation energy of CdPS_3_ was calculated by linearly fitting the logarithmic electrical conductivity as a function of 10,000/*T* using the following equation:

*σ* = *σ*_0_ exp (−*E*_t_/*k*_B_*T*) (3)

where *σ*_0_ is the pre-exponential factor (S m^−1^), *E*_t_ is the activation energy (eV), *k*_B_ is the Boltzmann constant and *T* is the absolute temperature (K) [37]. The pressure dependence of activation energy of CdPS_3_ is plotted in Figure S5. It is clear that the activation energies significantly diminished as the pressure was raised from 37.2 to 49.7 GPa, indicating the reduced energy barrier for ion hopping. When the pressure approached 52.1 GPa, the activation energy decreased to zero, hinting at the occurrence of metallization. Taken together, our variable-temperature electrical conductivity results confirmed that the semiconducting CdPS_3_ converted into the metallic state at 52.1 GPa.

### 2.4. Phase Diagram of MPS_3_ Under Extreme Conditions

**Figure 6:**
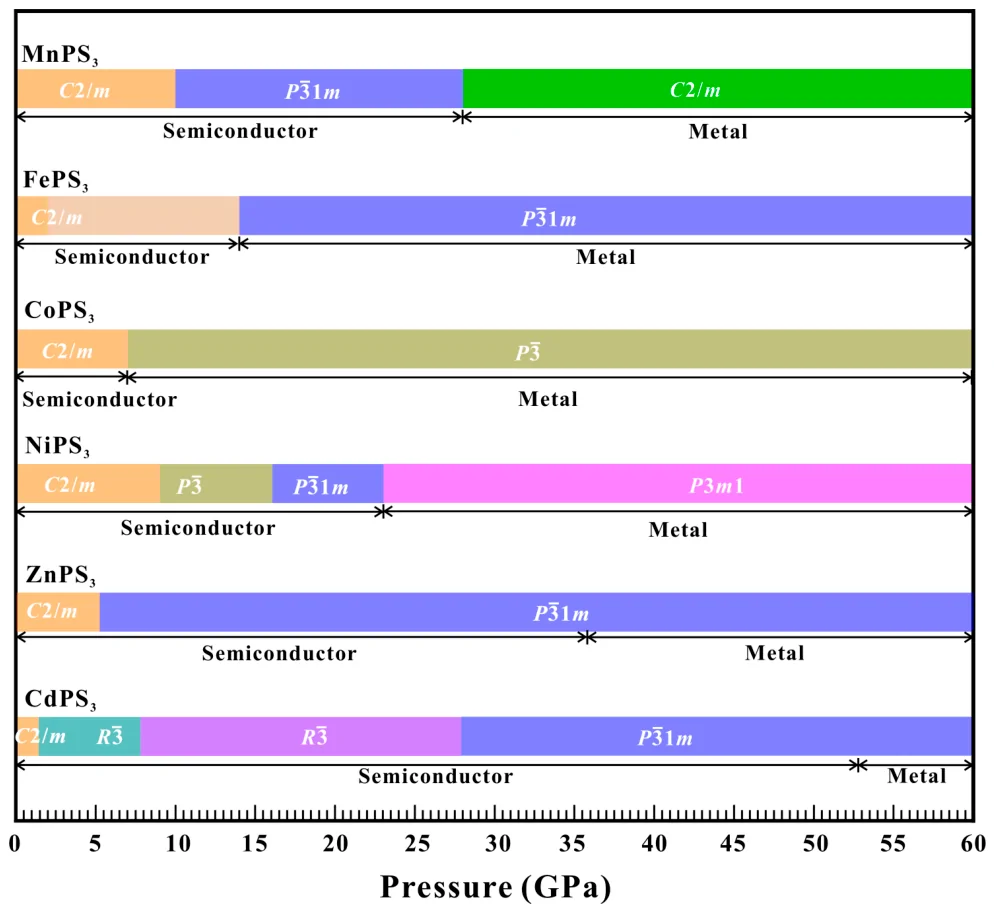
High-pressure phase diagram of the MPS_3_ family (M = Mn, Fe, Co, Ni, Zn and Cd) within the pressure range of 0–60.0 GPa reported in this work and previous studies.

Based on our high-pressure Raman scattering and electrical conductivity results, CdPS_3_ probably endured a succession of structural transitions from *C*2/*m* to *R*$\bar{3}$ to isostructural *R*$\bar{3}$ to *P*$\bar{3}1$*m* phases at the respective pressures of 1.4(5), 7.8(5) and 29.8(11) GPa, followed by a metallization at 51.3(8) GPa under the non-hydrostatic condition. In combination with our and prior studies, the phase diagram of the MPS_3_ family (M = Mn, Fe, Co, Ni, Zn and Cd) was constructed within the pressure range of 0–60.0 GPa, as depicted in Figure 6 [6,9,10,11,17,18,19,26]. From the structural perspective, MPS_3_ contains covalent, ionic and vdW bonds. Each phosphorus atom is covalently bonded to three sulfur atoms in an irregular tetrahedral geometry, and further, cadmium atoms are ionically bonded to the [P_2_S_6_] units. The coexistence of covalent and ionic bonds leads to the dense and rigid layers, and the layers are weakly coupled with each other by vdW forces along the *c* axis [8]. Within the MPS_3_ family, the overall structure of each layer is approximately the same, whereas the symmetry and stacking arrangements of individual layers in bulk crystals are governed by the specific cations [38]. Upon compression, the interlayer sliding (or interlayer distortion) along different crystallographic directions may lead to the diverse stacking sequences and structural transitions in the MPS_3_ system (M = Mn, Fe, Co, Ni, Zn and Cd). Despite the common structural building blocks of the [P_2_S_6_]^4−^ anion sublattice, each member in the MPS_3_ family hosts different structural transition paths and pressures due to the specific metal cations.

In combination with our and previous studies, the pressure-driven metallization should be a universal trend in the MPS_3_ family (M = Mn, Fe, Co, Ni, Zn and Cd) [6,9,10,11,17,18,19,26]. To our knowledge, CdPS_3_ has the highest metallization pressure in the MPS_3_ family, which was probably correlated with the number of electrons in the outermost shell of the metal elements. In other words, the metallization pressure of the *d*^10^ closed-shell cations (e.g., Cd^2+^, Zn^2+^, etc.) is higher than that of the partially filled *d* cations (e.g., Fe^2+^, Mn^2+^, Ni^2+^, etc.). At atmospheric pressure, the fully filled *d* electrons in Cd^2+^ and Zn^2+^ do not contribute their electrical conductivities and thus manifest as wide-bandgap semiconductors. In compounds with partially filled *d* cations, the electrons contribute to electrical conduction, giving rise to narrow-bandgap energies. In addition, the partially occupied *d* electrons often exhibit Jahn–Teller distortion, resulting in local distortions in their octahedral coordination and localized *d* electronic states. When applying pressure, lattice contraction may suppress the Jahn–Teller effect and thus contribute to a more symmetric coordination environment. The suppression of Jahn–Teller distortion and the resulting delocalization of *d* electrons may facilitate the occurrence of metallization in partially occupied *d* compounds under high pressure [39,40]. On the other hand, since the 4*d* shell of Cd^2+^ is completely full, no significant correlations were observed in CdPS_3_ due to the Coulomb repulsion. In the half-filled cations (e.g., Fe^2+^, Mn^2+^, Ni^2+^, etc.), the bandgap closure leads to the enhanced screening of the on-site Coulomb repulsion, which in turn promotes the rate of bandgap closure. Consequently, the core electrons of Cd^2+^ may account for the different structural transition paths and pressures together with the robust electronic stability of CdPS_3_ under compression. Apart from the fundamental properties, our high-pressure research indicated that CdPS_3_ holds great promise for applications in pressure-sensing functional materials, photodetectors and spintronic devices. Due to the high sensitivity of its electrical conductivity to pressure at 29.8–51.3 GPa, CdPS_3_ has potential application in pressure-sensing functional materials [41]. As an ideal candidate in solar-blind ultraviolet photodetectors, the pressure-induced metallization of CdPS_3_ provides an effective method to optimize its photoresponse for high-performance and low-noise ultraviolet detectors [42]. CdPS_3_ is of two-dimensional (2D) antiferromagnetic ordering and its physicochemical properties can be effectively modulated by pressure, which offers a new platform for next-generation spintronic devices [20].

## 3. Materials and Methods

### 3.1. Sample Preparation and Characterizations

**Figure 7:**
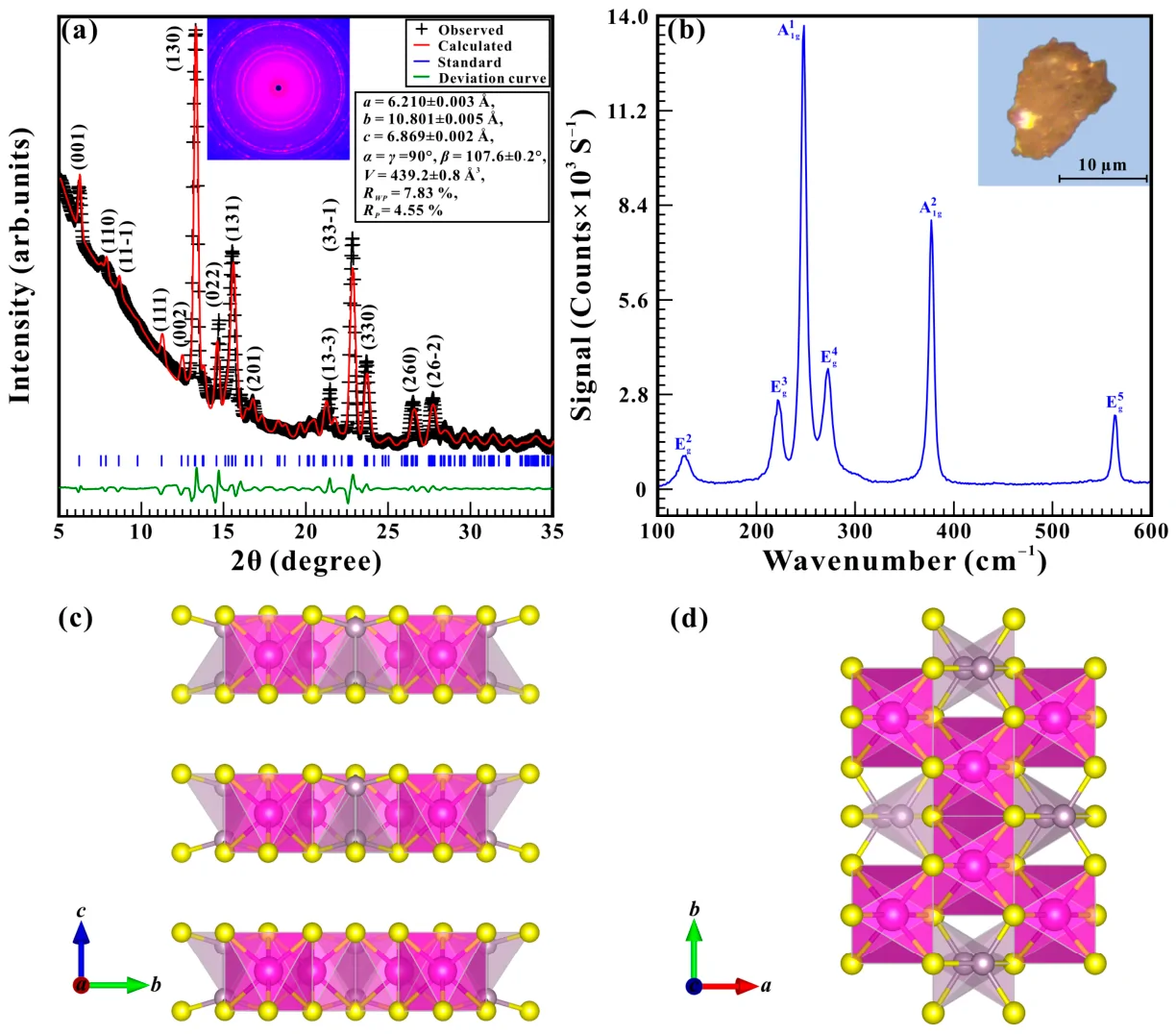
Structural characterizations of experimental sample under ambient conditions. (**a**) The representative XRD profile. Symbols: black crosses: observed pattern; red solid line: calculated pattern; blue vertical lines: diffraction peak positions of monoclinic CdPS_3_ with the space group of *C*2/*m*; green solid line: deviation between the observed and calculated patterns. The inset is 2D diffraction rings and the relevant lattice parameters of CdPS_3_. (**b**) Raman spectrum of the initial specimen. Inset: optical image of the experimental specimen with the scale bar of 10 μm. (**c**,**d**) Schematic representations of the crystalline structure of CdPS_3_ using the front and top views, respectively. Herein, the purple, gray and yellow balls stand for cadmium, phosphorus and sulfur atoms, respectively.

CdPS_3_ powders with yellow appearance (see the inset of Figure 7b) were obtained from Nanjing MKNANO Tech. Co., Ltd. (Nanjing, China) (www.mukenano.com, accessed on 7 August 2026). The crystalline structure of the pristine sample was determined using a Rigaku NanoPix-We diffractometer with Mo *Kα* radiation (X-ray wavelength being 0.7107 Å) operated at 40 kV voltage and 20 mA current. Rietveld refinement of the collected X-ray diffraction (XRD) pattern was conducted using the General Structure Analysis System (GSAS-II) program so as to identify the crystal structure and determine the lattice parameters of the starting sample. The Bragg diffraction peaks in Figure 7a can be well indexed by the monoclinic CdPS_3_ with the space group of *C*2/*m*, yielding the lattice parameters of *a* = 6.210 ± 0.003 Å, *b* = 10.801 ± 0.005 Å, *c* = 6.869 ± 0.002 Å, *α* = *γ* = 90°, *β* = 107.6 ± 0.2°, and *V* = 439.2 ± 0.8 Å^3^ under ambient conditions. From Figure 7b, the initial sample exhibits multiple Raman peaks at the positions of 127.0, 221.5, 248.3, 272.5, 377.3 and 562.7 cm^−1^. In brief, our XRD and Raman spectra are consistent with prior results on CdPS_3_ and thus confirmed the structural integrity of the experimental specimen [4,5,6,7,8,19,20,30,43,44,45,46]. Meanwhile, the schematic illustrations on the crystalline structure of CdPS_3_ from the front and top views are displayed in Figure 7c and d, respectively.

### 3.2. High-Pressure Raman Spectroscopy Experiments

High-pressure Raman scattering measurements of CdPS_3_ were carried out by the utilization of a symmetric DAC with a culet diameter of 200 μm and bevel angle of 10°. The experimental sample, PTM and ruby chips acting as the pressure gauge were sealed into a drilled hole of 100 μm diameter in a pre-indented T-301 stainless steel gasket, which was compressed from an initial thickness of 200 μm to roughly 50 μm at ~10.0 GPa [47]. In the high-pressure Raman spectroscopy experiments of CdPS_3_, the hydrostatic condition was achieved by helium using gas-loading apparatus and no PTM was employed for the non-hydrostatic condition. The uncertainties of pressure calibration under non-hydrostatic and hydrostatic environments were less than 6% and 3%, respectively [48]. Raman spectra of CdPS_3_ were collected by the Renishaw inVia spectrometer attached with two different excitation sources of the argon-ion laser (*λ* = 514.5 nm) and the diode laser (*λ* = 785.0 nm) in backscattering geometry. All gathered Raman spectra were processed by the initial baseline subtraction and subsequent Lorentzian fitting to extract the Raman peak positions and FWHMs of CdPS_3_ at high pressures.

### 3.3. High-Pressure Electrical Conductivity Measurements

As for the electrical conductivity experiments of CdPS_3_, an insulating sample chamber was prepared by pre-compressing a T-301 stainless steel gasket and then drilling a 180 µm diameter central hole. A powder mixture of boron nitride and epoxy was intercalated between the gasket and culet, followed by manufacturing another 100 µm diameter sample chamber. Insulating cement was applied to the rest of gasket for complete electrical insulation. No PTM was used to prevent possible interferences in electrical conductivity measurements. Two pieces of platinum electrodes with a thickness of ~2 um were in direct contact with the upper and lower surfaces of the sample. Impedance measurements were implemented with a Solartron-1260 impedance/gain phase analyzer in the frequency range of 10^0^–10^7^ Hz with a signal voltage of 3000 mV. In variable-temperature electrical conductivity measurements, the cryogenic temperature was realized by the evaporation of liquid nitrogen, which was monitored by a *K*-type thermocouple attached to the sidewall of the anvil. To check the reproducibility, the high-pressure Raman scattering and electrical conductivity experiments of CdPS_3_ under different hydrostatic environments were performed three times. More details on sample assembly and experimental procedures are described elsewhere [23,24,25,26,32,33,34,35,36].

## 4. Conclusions

In this work, we presented a systematic high-pressure investigation on the structural evolution and electrical transport properties of CdPS_3_ by means of Raman spectroscopy and electrical conductivity measurements. CdPS_3_ transformed from *C*2/*m* to *R*$\bar{3}$ phases at 1.4(5) GPa, then to an isostructural *R*$\bar{3}$ phase at 7.8(5) GPa, and finally to a *P*$\bar{3}1$*m* phase at 29.8(11) GPa, followed by a metallization at 51.3(8) GPa under non-hydrostatic pressurization. Nevertheless, the *R*$\bar{3}$-to-*P*$\bar{3}1$*m* structural transition and metallization of CdPS_3_ were postponed by ~2.0 GPa under the hydrostatic condition due to the influence of deviatoric stress. Upon depressurization, the reversible Raman spectra and electrical conductivity magnitude provided convincing evidence on the reversibility of structural transformations under different hydrostatic environments. This work on CdPS_3_ facilitates the exploration of high-pressure physicochemical characteristics and further development of potential applications for other layered vdW materials.

## Data Availability

The data that support the findings of this study are available from the corresponding author upon reasonable request.

## Supplementary Materials

The following supporting information can be downloaded at https://www.mdpi.com/article/10.3390/molecules31162760/s1. Figure S1: High-pressure Raman scattering patterns of CdPS_3_ under hydrostatic condition; Figure S2: Pressure-dependent Raman FWHM relations for the $E_{g}^{2}$, $E_{g}^{3}$, $A_{g}^{1}$ and $E_{g}^{5}$ modes under the hydrostatic condition; Figure S3: High-pressure Raman spectra of CdPS_3_ under the non-hydrostatic condition using a 785.0 nm excitation source; Figure S4: The evolution of relaxation frequency as a function of pressure; Figure S5: The evolution of activation energy as a response of pressure on CdPS_3_ within the pressure range of 37.2–52.1 GPa; Table S1: Pressure-dependent Raman shift (d*ω*/d*P*, cm^−1^ GPa^−1^) for CdPS_3_ upon pressurization under different hydrostatic environments; Table S2: Pressure-dependent Raman FWHM (d*F*/d*P*, cm^−1^ GPa^−1^) for CdPS_3_ upon pressurization under different hydrostatic environments; Table S3: The pressure dependence of activation energy (d*H*/d*P*, meV GPa^−1^) for CdPS_3_.
