# Supplementary material for: Experimental Observations on the Structural Transitions and Metallization in the Two-Dimensional Layered Compound of Cadmium Phosphide Sulfide Under Extreme Conditions
Source: Molecules. 2026 Aug 8;31(16):2760. doi: 10.3390/molecules31162760 (PMC13515235; doi:10.3390/molecules31162760)
Supplement: Supplementary file 1 [file molecules-31-02760-s001.zip › molecules-4406143-supplementary.pdf]

Supplementary materials

Experimental observations on the structural transitions and metallization in the two-dimensional layered compound of cadmium phosphide sulfide under extreme conditions

Xinyu Zhang[a], Lidong Dai[b*], Haiying Hu[b], Ziqiang Xu[b], Juxiang Shao[a], Hongchun Luo[a], Jiajun Zhu[a], Zhongying Mi[c], Tao Wang[a], Miao Ren[a], Yu Gao[a], Meiling Hong[a]

AFFILIATIONS

[a]Key Laboratory of Computational Physics of Sichuan Province, College of Mathematics and Physics, Yibin University, Yibin 644007, China;

[b]School of Physics and Electronic Science, Guizhou Normal University, Guiyang 550025, China

[c]Key Laboratory of High-temperature and High-pressure Study of the Earth's Interior, Institute of Geochemistry, Chinese Academy of Sciences, Guiyang 550081, China

[*]Author to whom correspondence should be addressed: dailidong@gznu.edu.cn

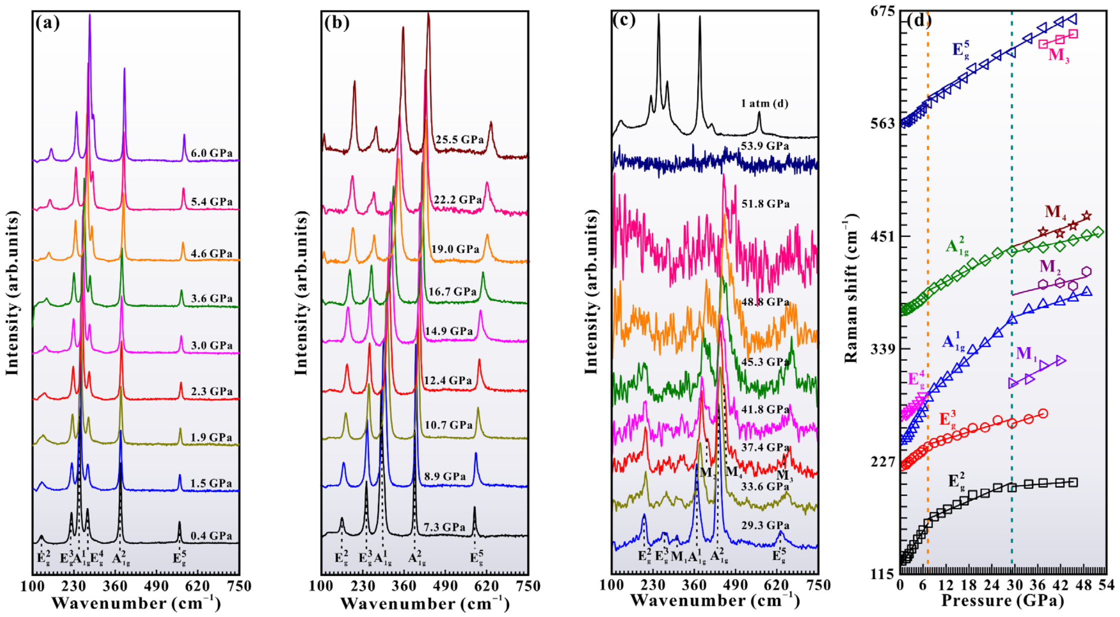

Figure S1. High-pressure Raman scattering patterns of $CdPS_3$ under hydrostatic conditions. (a–c) The results from 0.4 GPa to 53.9 GPa upon compression and the decompressed specimen under ambient conditions. Herein, the symbol of d denotes depressurization. (d) The corresponding variation of Raman shifts as a function of pressure. The errors in Raman shifts are within the size of the symbols.

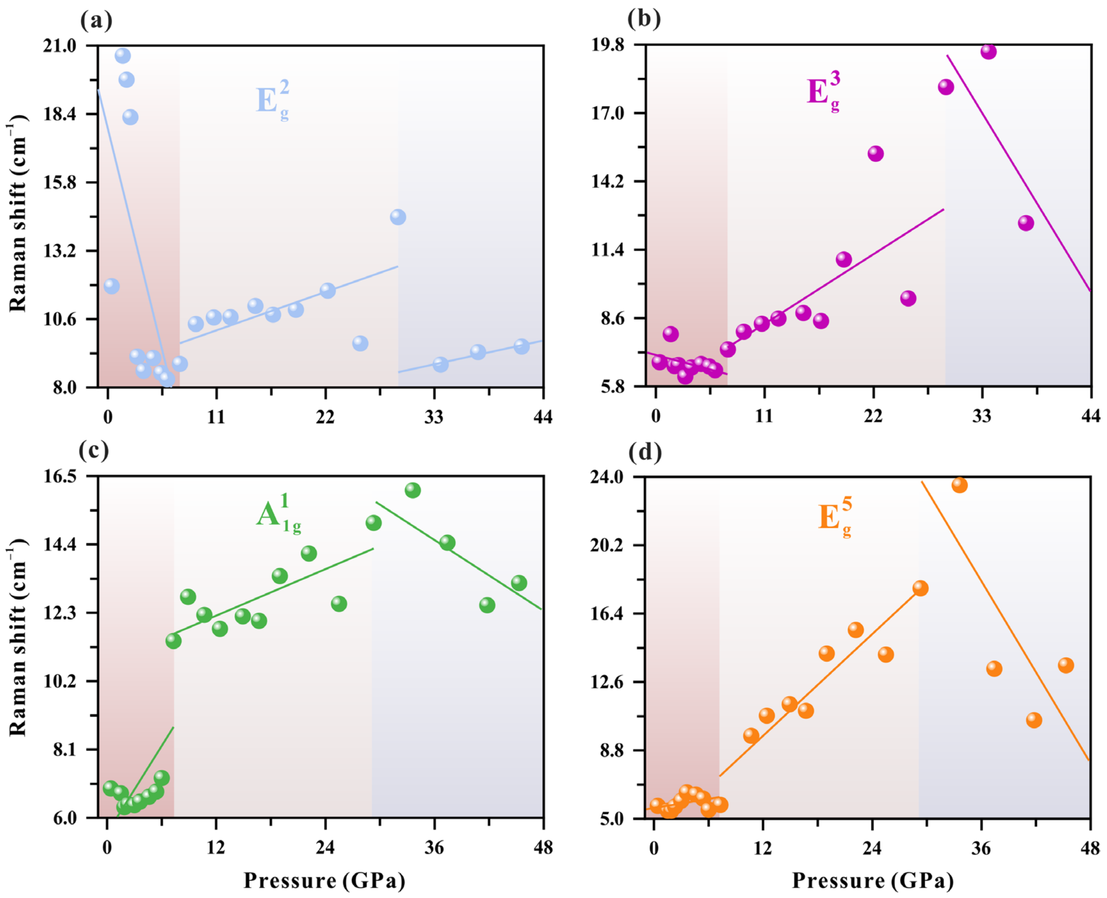

Figure S2. Pressure-dependent Raman FWHM relations for the (a) E_g^2, (b) E_g^3, (c) A_g^1 and (d) E_g^5 modes under hydrostatic conditions, respectively. The errors in Raman FWHMs are within the size of the symbols.

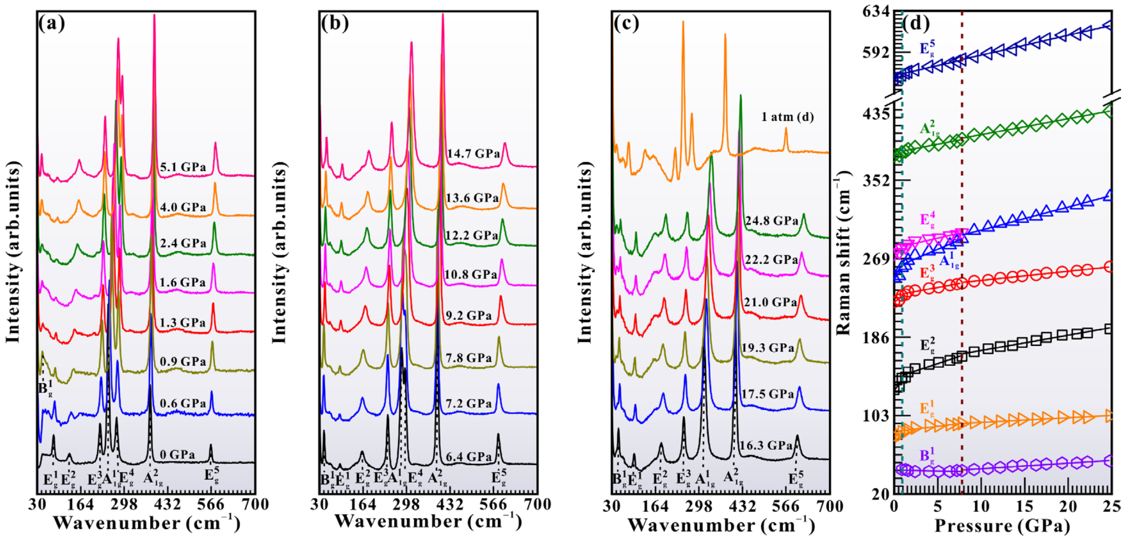

Figure S3. High-pressure Raman spectra of $CdPS_3$ under non-hydrostatic conditions using a 785 nm excitation source. (a–c) The results from ambient pressure to 24.8 GPa upon compression and the decompressed specimen at atmospheric pressure. Herein, the symbol of d denotes depressurization. (d) The related evolution of Raman shifts as a function of pressure. The errors in Raman shifts are within the size of the symbols.

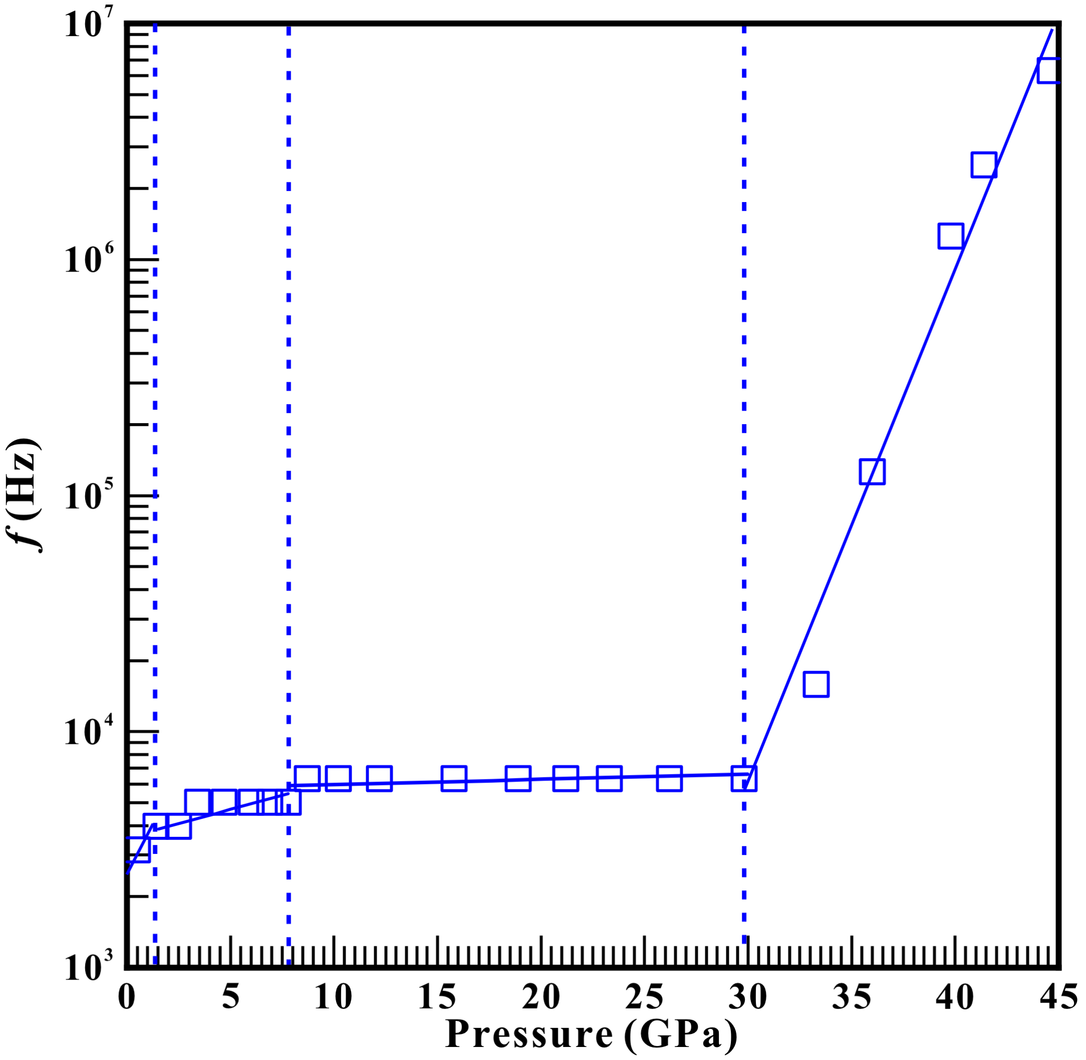

Figure S4. The evolution of relaxation frequency as a function of pressure at room temperature for $CdPS_3$. Herein, the signal of *f* refers to relaxation frequency. The errors in relaxation frequencies are within the size of the symbols.

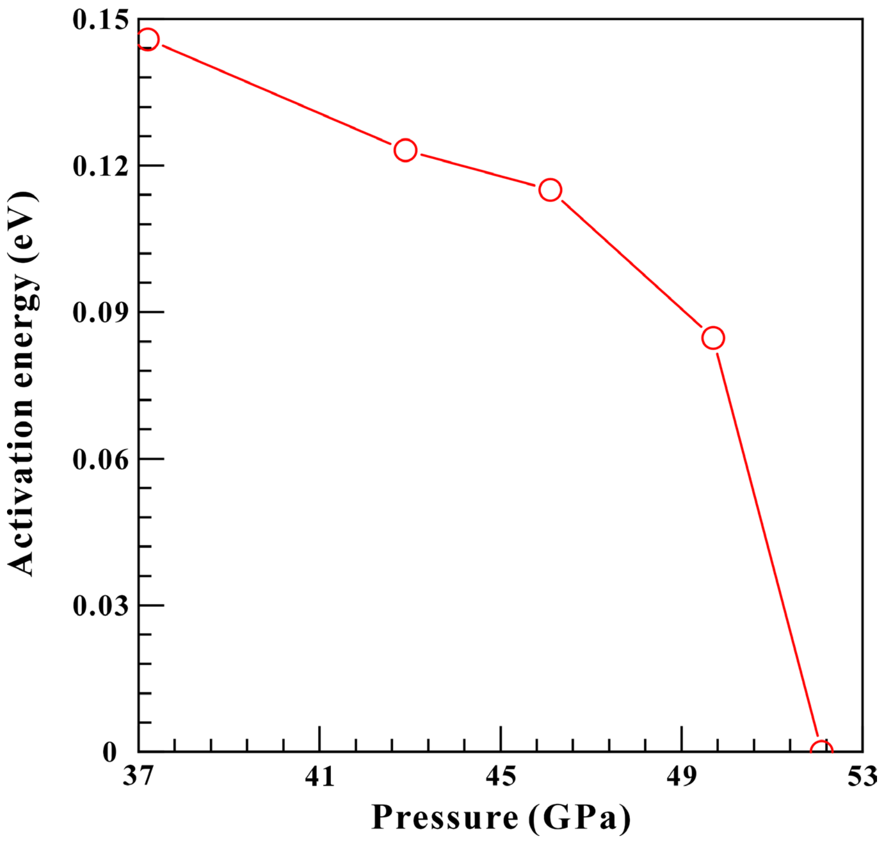

Figure S5. The evolution of activation energy as a response of pressure on $CdPS_3$ within the pressure range of 37.2–52.1 GPa.

Table S1. Pressure-dependent Raman shift (dω/dP, cm^{-1} GPa^{-1}) for $CdPS_3$ upon pressurization under different hydrostatic environments. Herein, the signals of ω (cm^{-1}) and P (GPa) represent Raman frequency and pressure, respectively.

Pressure conditions	Pressure range (GPa)	ω (cm^{-1})	dω/dP (cm^{-1} GPa^{-1})	ω (cm^{-1})	dω/dP (cm^{-1} GPa^{-1})
		127.9 (E_g^2)	6.13	222.0 (E_g^3)	3.19
	0.6–7.8	248.2 (A_{1g}^1)	6.59	272.7 (E_g^4)	3.91
		377.6 (A_{1g}^2)	2.66	563.5 (E_g^5)	3.15
		167.1 (E_g^2)	1.71	242.1 (E_g^3)	1.14
	7.8–27.9	291.6 (A_{1g}^1)	3.36	–	
Non-hydrostatic		395.5 (A_{1g}^2)	1.90	584.6 (E_g^5)	2.38
		202.8 (E_g^2)	0.32	263.1 (E_g^3)	1.18
		361.2 (A_{1g}^1)	1.54	–	
	27.9–50.8	433.3 (A_{1g}^2)	0.86	632.8 (E_g^5)	1.06
		298.4 (M_1)	1.81	385.5 (M_2)	2.10
		630.2 (M_3)	1.03	453.4 (M_4)	3.91
		128.1 (E_g^2)	5.85	222.5 (E_g^3)	2.97
	0.4–7.3	247.3 (A_{1g}^1)	6.46	272.8 (E_g^4)	3.52
		376.9 (A_{1g}^2)	2.59	563.2 (E_g^5)	2.98
		165.5 (E_g^2)	1.64	242.0 (E_g^3)	1.15
	7.3–29.3	290.3 (A_{1g}^1)	3.45	–	
Hydrostatic		394.4 (A_{1g}^2)	1.97	583.4 (E_g^5)	2.59
		201.0 (E_g^2)	0.29	264.9 (E_g^3)	1.22
		367.6 (A_{1g}^1)	1.29	–	
	29.3–51.8	436.0 (A_{1g}^2)	0.81	633.5 (E_g^5)	2.06
		304.6 (M_1)	1.96	402.2 (M_2)	0.93
		641.7 (M_3)	1.31	455.2 (M_4)	1.43

Table S2. Pressure-dependent Raman full width at half maximum (FWHM) (d*F*/d*P*, cm^{-1} GPa^{-1}) for $CdPS_3$ upon pressurization under different hydrostatic environments. Herein, the signals of ω (cm^{-1}), F (cm^{-1}) and P (GPa) stand for Raman frequency, Raman FWHM and pressure, respectively.

Pressure conditions	Pressure range (GPa)	ω (cm^{-1})	F (cm^{-1})	d*F*/d*P* (cm^{-1} GPa^{-1})	ω (cm^{-1})	F (cm^{-1})	d*F*/d*P* (cm^{-1} GPa^{-1})
Non-hydrostatic	0.6–7.8	127.9 (E_g^2)	13.91	–0.67	222.0 (E_g^3)	9.0	–0.21
		248.2 (A_{1g}^1)	6.6	0.50	563.5 (E_g^5)	6.29	0.41
	7.8–27.9	167.1 (E_g^2)	12.07	0.039	242.1 (E_g^3)	9.47	0.054
		291.6 (A_{1g}^1)	14.48	0.13	584.6 (E_g^5)	11.74	0.21
	27.9–40.4	202.8 (E_g^2)	9.37	0.96	263.1 (E_g^3)	21.97	–0.54
		361.2 (A_{1g}^1)	19.52	–0.44	632.8 (E_g^5)	30.11	–1.66
Hydrostatic	0.4–7.3	128.1 (E_g^2)	11.86	–1.54	222.5 (E_g^3)	6.80	–0.11
		247.3 (A_{1g}^1)	6.91	0.45	563.2 (E_g^5)	5.72	0.082
	7.3–29.3	165.5 (E_g^2)	8.89	0.13	242.0 (E_g^3)	7.32	0.26
		290.3 (A_{1g}^1)	11.43	0.12	583.4 (E_g^5)	5.77	0.51
	29.3–45.3	201.0 (E_g^2)	14.48	0.082	264.9 (E_g^3)	18.07	–0.67
		367.6 (A_{1g}^1)	15.07	–0.18	633.5 (E_g^5)	17.81	–0.84

Table S3. The pressure dependence of activation energy (d*H*/d*P*, meV GPa^{-1}) for $CdPS_3$ within four distinct pressure ranges of 0.5–1.4 GPa, 1.4–7.8 GPa, 7.8–29.8 GPa and 29.8–44.6 GPa, respectively. Wherein, the signals of *H* (meV) and *P* (GPa) represent activation energy and pressure, respectively.

Pressure range (GPa)	d*H*/d*P* (meV GPa^{-1})	Error (%)
0.5–1.4	–6.48	0.44
1.4–7.8	–0.94	0.57
7.8–29.8	–0.10	0.17
29.8–44.6	–12.83	2.10
